# Fractal analysis of left ventricular trabeculae in hypertensive patients with heart failure: a 3.0 T cardiac magnetic resonance study

**DOI:** 10.3389/fcvm.2025.1697453

**Published:** 2025-11-27

**Authors:** Yunxia Xie, Zhenyi Zhao, Kun Deng, Haibo Ren, Sisi Yu, Ziyan Feng, Jiahui Zhang, Hui Liu, Lianggeng Gong

**Affiliations:** 1 Department of Radiology, The Second Affiliated Hospital, Jiangxi Medical College, Nanchang University, Nanchang, China; 2 Intelligent Medical Imaging of Jiangxi Key Laboratory, Nanchang, China; 3 Artificial Intelligence, The School of Computer and Artificial Intelligence, Zhengzhou University, Zhengzhou, China

**Keywords:** heart failure, hypertension, left ventricular myocardial trabecular, fractal analysis, magnetic resonance imaging

## Abstract

**Background:**

Endocardial trabecular hyperplasia due to hemodynamic stress reflects phenotypic variability in disease progression. Employing fractal analysis, this study quantified left ventricular (LV) myocardial trabecular complexity in hypertensive patients with and without heart failure (HF) to evaluate its diagnostic utility for HF.

**Methods:**

This study retrospectively enrolled 146 hypertensive patients (77 with HF, 69 without), grouped into HTN-HF (*n* = 77) and HTN non-HF (*n* = 69); additionally, 34 healthy controls were recruited. Clinical data and cardiac MRI parameters were compared. Fractal dimension (FD) values were calculated on the LV short-axis cine images using fractal analysis. Logistic regression analysis was performed to determine predictors.

**Results:**

Five fractal dimensions were derived: global FD, along with mean/maximal apical FD and mean/maximal basal FD. Compared with healthy controls, HF patients showed significantly elevated left ventricular fractal dimensions (all *P* < 0.001). Moreover, these fractal dimensions exhibited significant differences between the HTN-HF patients and HTN non-HF patients, except for maximal basal FD. The univariate logistic regression revealed that global FD, mean/maximal apical FD and mean basal FD emerged as significant independent predictors (OR: 1.170,1.121,1.070, and 1.088, *P* < 0.05). Furthermore, integration of fractal dimensions enhanced calibration and diagnostic accuracy of the model. (AUC: 0.877).

**Conclusions:**

CMR fractal analysis provides a feasible technique for quantifying LV myocardial trabecular complexity in hypertensive heart failure patients. In conclusion, our study demonstrates the potential of fractal analysis to provide incremental diagnostic value for heart failure within the hypertensive population. Integration of FD into clinical diagnostic models may enhance diagnostic performance.

## Introduction

Hypertension (HTN) remains a significant global public health challenge, serving as a critical predisposing factor for heart failure (HF) and being strongly associated with elevated morbidity and mortality rates (1–3). Prolonged chronic hypertension imposes excessive burden on the left ventricle (LV), lending to left ventricular remodeling and ultimately evolving into hypertensive heart failure (4). Studies indicate that cardiac remodeling in these patients involves not only structural changes but also functional imbalance (5, 6).Therefore, early diagnosis of heart failure is of great significance for delaying cardiac remodeling and improving prognosis. However, the clinical manifestations of hypertensive HF are often nonspecific and may overlap with symptoms of HTN itself. This can lead to a lack of awareness, particularly in middle-aged and older populations, resulting in delayed diagnosis and poor outcomes.

Cardiac magnetic resonance (CMR) imaging has emerged as the gold standard for evaluating cardiac remodeling due to its non-invasiveness, high soft tissue resolution, and precise quantification capabilities (7, 8). Notably, CMR enables clear delineation of the complex anatomical features of the ventricular trabeculae. Fractal analysis, an emerging technique, offers a new quantitative perspective. This technique objectively describes the geometric complexity of myocardial trabeculae by calculating the fractal dimension (FD) (9, 10). Research suggests that abnormal trabecular proliferation may reflect early myocardial mechanical disturbances, even preceding traditional heart function abnormalities (11). Additionally, Sigvardsen et al. demonstrated an independent correlation between increased LV trabeculae and a higher incidence of major adverse cardiac events (12). A previous study revealed that increased LV trabeculae are associated with reduced LV torsion and untwisting rates (13), offering new insights into the pathophysiology of HF. Combining fractal analysis with CMR presents a promising approach for detecting subclinical cardiac remodeling.

However, the value of this innovative method in hypertensive patients with HF remains unclear. Existing research primarily focused on ischemic heart disease (14, 15), overlooking the broader HTN population. Hence, the present study adopts a cross-sectional design to systematically explore the additive diagnostic potential of CMR combined with fractal analysis in hypertensive HF. The fractal dimension of myocardial trabeculae, obtained through cardiac imaging, is hypothesized to reflect subtle structural changes linked to the onset and progression of HF, offering new perspectives for diagnosis and prevention.

## Methods

### Study population

This retrospective cross-sectional study included consecutive HTN patients who underwent CMR imaging at the Second Affiliated Hospital of Nanchang University between January 2018 and May 2024. Hypertension (HTN) was defined as an office systolic blood pressure (SBP) ≥ 140 mmHg and/or office diastolic blood pressure (DBP) ≥ 90 mmHg on at least two separate occasions, or a documented history of hypertension with ongoing antihypertensive medication (16). Next, HTN patients meeting the following criteria were included in the HF group: (I) LVEF < 50% or LVEF ≥50% but with objective evidence as the 2021 ESC HF guideline (17), requiring the presence of at least two functional and/or structural abnormalities (E/e' ratio > 9, left atrial enlargement, or left ventricular hypertrophy); (II) exhibiting the cardinal symptoms and/or signs of HF, such as dyspnea, peripheral edema, and fatigue; (III) NT-proBNP ≥125 pg/mL or BNP ≥35 pg/mL (17). In this study, HTN-HF patients refer to those with coexisting hypertension and heart failure.

Exclusion criteria for the effects of other heart diseases on the heart were as follows: (I) any condition causing myocardial hypertrophy such as hypertrophic cardiomyopathy, amyloidosis, Fabry disease, or athlete's heart; (II) severe arrhythmias; (III) primary moderate or severe valvular heart disease; (IV) anemia or hyperthyroidism; (V) history of myocardial infarction or cardiac surgery; and (VI) inability to complete CMR examination or poor image quality. Patients with HTN not satisfying the HF criteria were allocated to the HTN non-HF group. Additionally, a group of individuals without HTN and with negative CMR findings from routine health check-ups were selected as healthy controls.

### CMR protocol and analysis

Cardiac magnetic resonance imaging (MRI) examinations were performed on all participants with a 3.0 T scanner and 8-channel phased-array cardiac coil. Cardiac short-axis view images were acquired by using a standard breath-hold steady-state free precession cine sequence. The scan range included the entire LV short-axis slices from the apex to the base. The scan parameters included: short-axis slice thickness 8 mm and 8–13 slices; section gap 3 mm; repetition time (TR) 3.9 ms; echo time (TE) 1.6 ms; matrix size 256 × 256 mm; field of view (FOV) 360 × 360 mm; flip angle 55°.

Cardiac VX software was used to quantify LV functional parameters. The CMR image analysis was performed by observers who were blinded to the clinical data and group identity of all subjects. The LV endocardial and epicardial boundaries were semi-automatically contoured in the end-systolic and end-diastolic phases at the short-axis cine, and the parameters of the cardiac function were obtained after making appropriate adjustments. LV function parameters, including LV ejection fraction (LVEF), LV end-diastolic volume (LVEDV), LV end-systolic volume (LVESV), stroke volume (SV), and maximal LV wall thickness (maximal LVWT), were recorded. LV end-diastolic volume index (LVEDVI) and LV end-systolic volume index (LVESVI) were subsequently computed by normalizing LVEDV and LVESV to body surface area (BSA) obtained with the Mosteller formula.

### Fractal analysis

**Figure 1:**
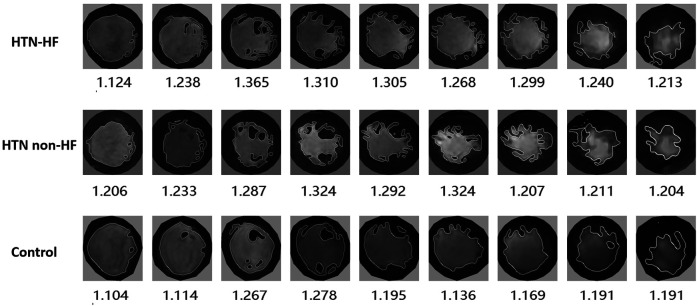
Demonstration of left ventricular fractal dimension (FD) in participants with HTN-HF, HTN non-HF and control groups. FD extraction steps: the endocardial border was extracted at end-diastole, and endocardial trabeculae FD was subsequently calculated using a box-counting approach.

Fractal analysis was performed using an open source code named FracAnalyse in MATLAB (MathWorks, Natick, Massachusetts, USA), which has been validated in previous studies (9, 15, 18–21). The region of interest was delineated at the endocardial border during the end-diastolic phase in every short-axis slice of the LV. Subsequently, the FDs were automatically calculated by the software employing the box-counting method. The analysis procedure included the following steps: (1) image binarization using multi-threshold single-level set algorithms to distinguish between the LV myocardium and the blood pool; (2) application of the Sobel filter to detect the endocardium and trabeculae; (3) extraction of the borders of the endocardium and computation of FDs for every slice (Figure 1); and (4) segmentation of papillary muscles to provide the final image edges for fractal analysis.

**Figure 2:**
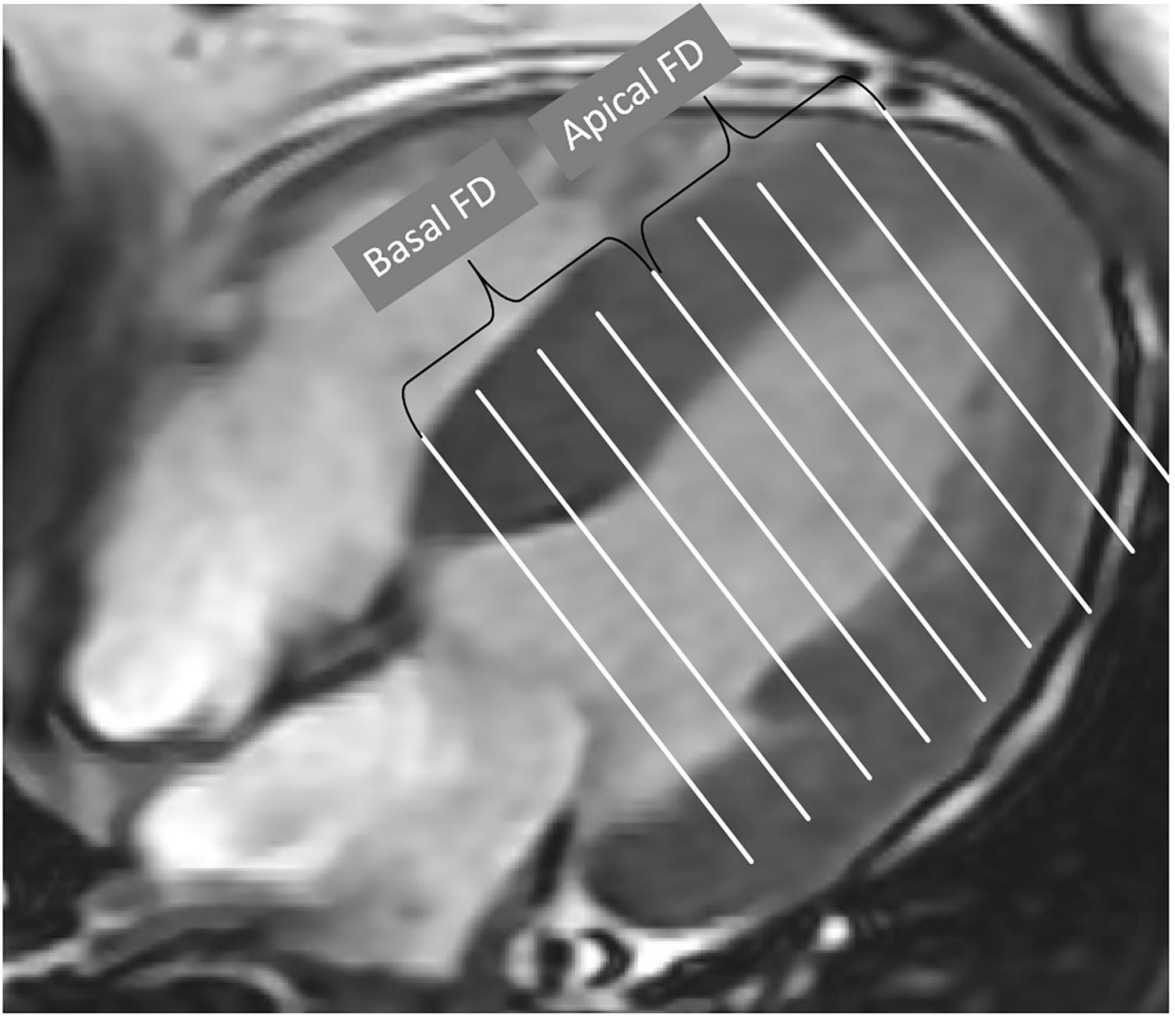
Global FD was defined as an average of all FD in all measured slices. Maximal basal FD and maximal apical FD were defined as the maximal value of the basal and apical slices of the ventricle. Mean basal FD and mean apical FD were defined as the average values of the corresponding slices.

The software finally calculated five non-dimensional FD parameters that reflect the complexity of the trabecular structure (Figure 2). The global FD was defined as an average of all FDs in all measured LV short-axis slices. The maximal basal FD and maximal apical FD were characterized as the peak FD values within the basal and apical ventricular slices, respectively. The mean basal FD and mean apical FD were ascertained as the average FD values within their corresponding slices. The CMR fractal analysis was performed by observers who were blinded to the clinical data and group identity of all subjects.

### Statistical analysis

Statistical analysis was conducted in SPSS 26.0 software. The normality of the data was assessed using the Shapiro–Wilk test. Normally distributed continuous variables appear as mean ± standard deviation (SD); non-normal data are reported as median with interquartile ranges (IQRs). Categorical variables display frequencies and percentages. For inter-group comparisons, normally distributed variables employed independent samples *t*-tests, whereas non-parametric variables used Mann–Whitney *U*-tests. Categorical variables were compared using the chi-square test or Fisher's exact test. Data comparisons among the three groups were performed using one-way analysis of variance (ANOVA) or the Kruskal–Wallis test. To ensure the robustness of the inter-group comparisons for the fractal dimension metrics against potential confounding by baseline demographic differences, analyses of covariance (ANCOVA) were additionally performed, adjusting for age and sex where appropriate.

The correlation between LV FD and cardiac function parameters was analyzed using Spearman correlation analysis. Univariate logistic regression analysis was employed to identify variables significantly associated with CMR findings. Variables with a *P* value < 0.1 in univariate analysis were included in multiple regression using forward selection. Multicollinearity was assessed using the variance inflation factor (VIF), and variables with a VIF > 5 were excluded from the final multivariate model. Traditional risk parameters were used as the baseline model; thereafter, LV function parameters and FD were added to create a risk nested model. The predictive performance of the models was assessed using receiver operating characteristic (ROC) curve analysis, and the DeLong test was performed to compare the area under the curve (AUC) values between models to determine the prognostic benefit improvement compared to the baseline model. All statistical tests were two-tailed, and a *P*-value < 0.05 was considered statistically significant.

### Intra-observer and inter-observer reproducibility

To ensure the inter-observer and intra-observer reproducibility of LV FDs, 30% of patient images from our study cohort were randomly selected for re-evaluation. Two CMR-experienced radiologists independently assessed LV FD using same datasets with mutual blinding. Intra-observer reproducibility was evaluated by a single radiologist measuring LV FD twice, a month apart. Inter- and intra-observer consistency was quantified using intraclass correlation coefficients (ICC).

### Ethical considerations

Approved by our hospital's institutional review board (IRB), this retrospective study adhered to the Declaration of Helsinki. The retrospective design of the investigation exempted the requirement for written informed consent.

## Results

### Demographic and baseline clinical characteristics

As shown in Table 1, a total of 171 HTN patients were enrolled in this study. Seven patients with hypertrophic cardiomyopathy, three with ischemic cardiomyopathy, five with myocardial infarction, three with valvular heart disease, one with severe arrhythmia, and six patients with poor short-axis cine sequence images were excluded. A final cohort of 146 patients was included in the study (mean age 51.03 ± 15.94 years, 79% male). Among these 146 patients, 77 HTN patients had HF (mean age 53.13 ± 17.23 years, 75% male), and 69 patients had HTN without HF (mean age 48.70 ± 14.16 years, 84% male). Additionally, 34 healthy participants (mean age 39.29 ± 16.99 years, 62% male) were selected as controls. Compared with the control group, the HTN non-HF group had greater age, a higher proportion of males, and higher BMI, SBP, and DBP (all *P* < 0.05). There were no significant differences between the HTN non-HF and HTN-HF groups in terms of sex, age, BMI, blood pressure, medical history, and pharmacological treatment (all *P* > 0.05). Compared with HTN non-HF group, those with HF were more likely to have New York Heart Association (NYHA) class II or higher symptoms [65 out of 77 patients [84%] vs. 23 out of 69 patients [33%]; *P* < 0.001].

**Table 1:** Clinical baseline characteristics and CMR parameters of participants.

Characteristics	HTN non-HF (*n* = 69)	HTN-HF (*n* = 77)	Controls (*n* = 34)	*P*
Demographics				
Age (years)	48.70 ± 14.11*	53.13 ± 17.23*	39.29 ± 16.99	<0.001
Males	58 (84.0)*	58 (75.3)*	21 (61.8)	<0.001
BMI (kg/m^2^)	26.62 ± 4.41*	26.49 ± 3.96*	23.22 ± 3.83	<0.001
BSA (m^2^)	1.87 ± 0.23*	1.82 ± 0.22*	1.75 ± 0.21	0.03
SBP (mm Hg)	156.59 ± 24.10*	152.16 ± 27.28*	121.65 ± 9.17	<0.001
DBP (mm Hg)	96.45 ± 20.12*	93.78 ± 23.68*	78.15 ± 5.57	<0.001
NYHA functional class				<0.001
I	46 (66.7)	12 (15.6)		
II	22 (31.9)	51 (66.2)		
III	1 (1.5)	12 (15.6)		
IV	0 (0)	2 (2.6)		
NYHA ≥ II	23 (33.33)	65 (84.42)^#^		
Medications				
ACEI/ARB	44 (63.8)	53 (68.8)		
Beta-Blocker,	21 (30.4)	37 (48.0)^#^		
Diuretics	17 (24.6)	27 (35.1)		
Calcium Channel Blocker	47 (68.1)	40 (51.9)^#^		
Statin	23 (33.8)	27 (35.5)		
Past Medical History				
Hypertension	69 (100.0)	77 (100.0)		
Smoking history	28 (40.6)	30 (39.0)		
Alcohol history	24 (34.8)	23 (29.9)		
Diabetes	15 (21.7)	16 (20.8)		
Dyslipidemia	39 (56.5)	48 (62.3)		
Atrial fibrillation	3 (4.3)	5 (6.5)		
CMR parameters				
LVEF, %	60.87 ± 7.92*	49.96 ± 14.91*^,#^	62.23 ± 4.77	<0.001
LVEDV (mL)	130.57 (103.00, 160.00)*	154.17 (118.63, 187.00)*^,#^	109.50 (90.50, 151.75)	<0.001
LVESV (mL)	53.09 ± 21.47	83.82 ± 48.44*^,#^	40.00 (32.00,59.00)	<0.001
SV (L/min)	80.98 (67.00, 97.00)	73.00 (56.25, 91.76)	67.500 (57.50, 91.75)	0.158
Maximal LVWT(mm)	13.000 (12.00, 16.00)*	14.00 (13.00, 16.00)*	8.500 (8.00,9.00)	<0.001
LVEDVI (mL/m^2^)	68.50 (60.56, 82.76)*	83.90 (68.78, 105.37)*^,#^	66.76 (53.87, 80.73)	<0.001
LVESVI (mL/m^2^)	25.87 (21.85, 32.08)*	39.21 (27.60, 55.46)*^,#^	24.54 (18.01, 33.12)	<0.001
LV FD parameters				
Global FD	1.235 ± 0.04*	1.258 ± 0.038*^,#^	1.189 ± 0.038	<0.001
Mean apical FD	1.222 ± 0.055*	1.255 ± 0.053*^,#^	1.173 ± 0.051	<0.001
Maximal apical FD	1.309 (1.269, 1.333)*	1.322 (1.278, 1.361)*^,#^	1.233 (1.198, 1.268)	<0.001
Mean basal FD	1.241 ± 0.042*	1.256 ± 0.043*^,#^	1.194 ± 0.035	<0.001
Maximal basal FD	1.339 ± 0.045*	1.345 ± 0.039*	1.282 ± 0.036	<0.001

Data are presented as mean ± SD or median (IQRs) or *n* (%). HTN, hypertension; HF, heart failure; BMI, body mass index; BSA, body surface area; SBP, systolic blood pressure; DBP, diastolic blood pressure; ACEI, angiotensin-converting enzyme inhibitor; ARB, angiotensin receptor blocker; NYHA, New York heart association; CMR, cardiac magnetic resonance; LVEF, LV ejection fraction; LVEDV, LV end-diastolic volume; LVESV, LV end-systolic volume; SV, stroke volume; LV, Left ventricular; FD, fractal dimension; LVEDVI, LV end-diastolic volume index; LVESVI, LV end-systolic volume index; Maximal LVWT, maximal LV wall thickness.

* *P* < 0.05 vs. Controls.

# *P* < 0.05 vs. HTN non-HF group.

### Left ventricular CMR parameters and fractal dimension parameters

All CMR parameters and LV FD parameters were presented in Table 1. Statistically significant differences in LVEF were found among the HTN-HF, HTN non-HF, and healthy groups (49.96 ± 14.91 vs. 60.87 ± 7.92 vs. 62.24 ± 4.77, all *P* < 0.05). In contrast, no significant differences in SV were found among the three groups (*P* = 0.158). As for LVEDV, the HTN-HF group and HTN non-HF group were both higher than the healthy group [all *P* < 0.05; 154.17 (118.63, 187.00) vs. 130.57 (103.00, 160.00) vs. 109.50 (90.00, 151.75)]. Moreover, the LVEDV in patients of HTN-HF was higher than the HTN non-HF group [154.17 (118.63, 187.00) vs. 130.57 (103.00, 160.00), *P* < 0.05]. The HTN-HF group had a higher LVESV compared with the HTN non-HF and control groups (all *P* < 0.05). Both the HTN-HF and HTN non-HF groups exhibited significantly higher maximal LVWT compared with the control group, while no significant difference was found between the HTN-HF and HTN non-HF groups in terms of maximal LVWT.

**Figure 3:**
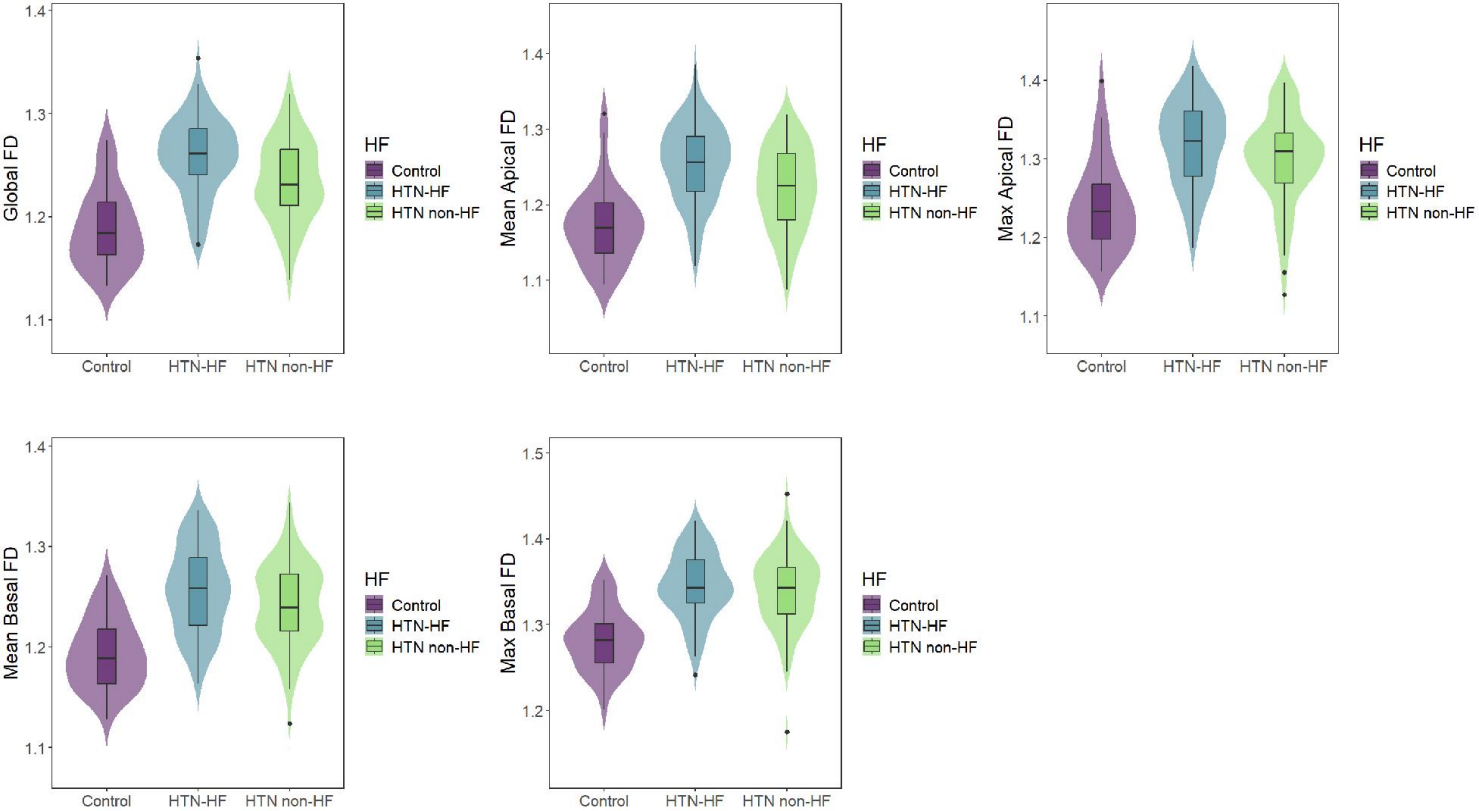
Comparisons of LV FDs among the HTN-HF, HTN non-HF, and control groups. LV, left ventricular; FD, fractal dimension; HTN, hypertension; HF, heart failure.

In terms of left ventricular fractal dimension, significant differences in global FD, mean apical FD, maximal apical FD, and mean basal FD were observed among the HTN-HF, HTN non-HF, and healthy groups (all *P* < 0.05), displaying an increasing trend (Table 1 and Figure 3). Crucially, these significant differences persisted after adjustment for differences in age and sex using analysis of covariance (ANCOVA) (for all four metrics, *P* < 0.001). However, no significant difference was found in maximal basal FD between the HTN-HF and HTN non-HF groups (*P* = 0.448; 1.345 ± 0.039 vs. 1.339 ± 0.045). This non-significance also remained after age and sex adjustment (*P* > 0.05). Nevertheless, both the HTN-HF and HTN non-HF groups had significantly higher maximal basal FD compared with the healthy control group (1.282 ± 0.036) (all *P* < 0.05). These comparisons against the healthy group for maximal basal FD also remained significant after adjustment (*P* < 0.05).

### Correlations between LV FD and ventricular function and clinical parameters

**Figure 4:**
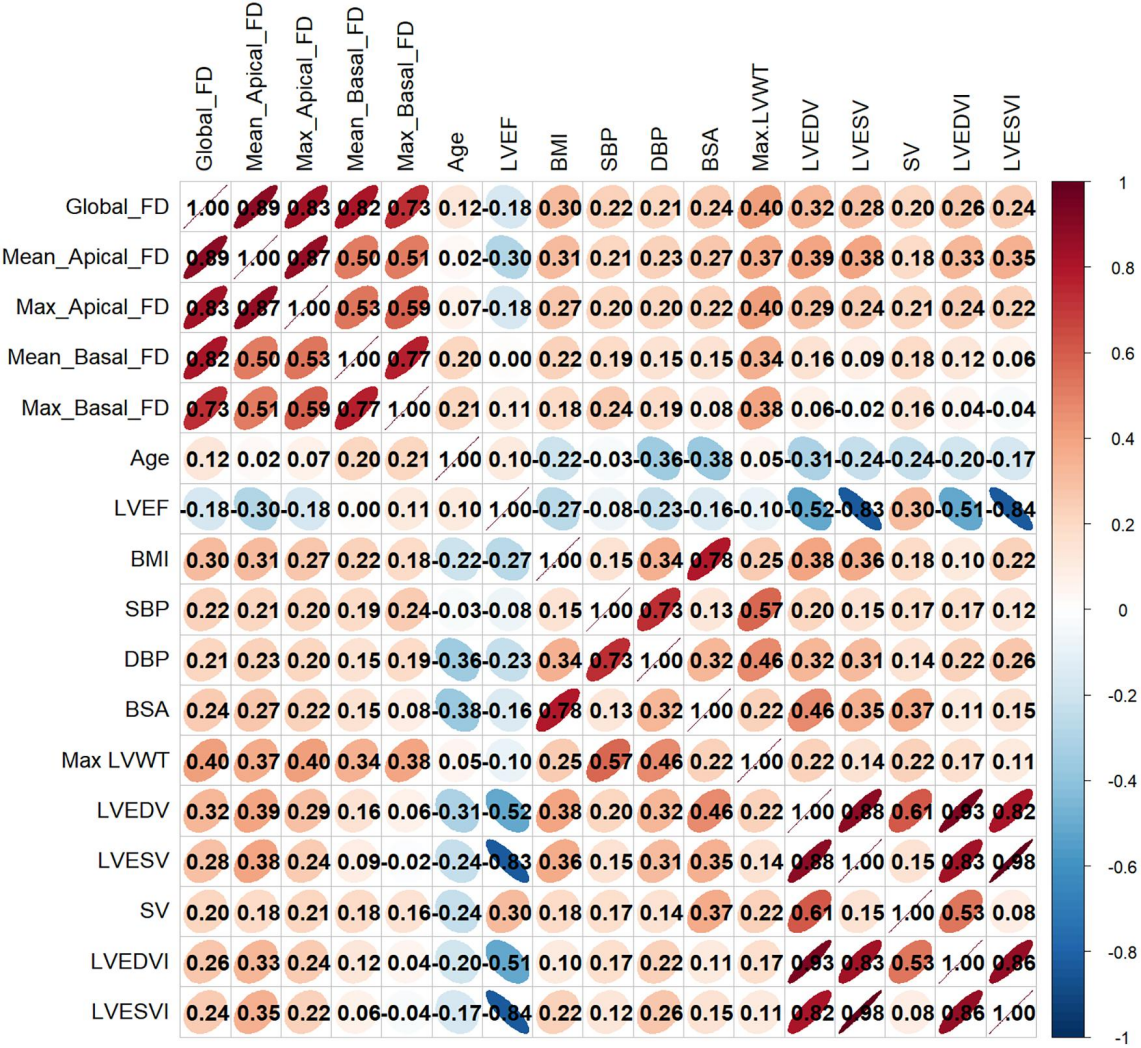
Correlation heatmap between LV FDs and LV functional parameters and clinical parameters. FD, fractal dimension; BMI, body mass index; BSA, body surface area; SBP, systolic blood pressure; DBP, diastolic blood pressure; LVEF, LV ejection fraction; LVEDV, LV end-diastolic volume; LVESV, LV end-systolic volume; SV, stroke volume; LV, left ventricular; LVEDVI, LV end-diastolic volume index; LVESVI, LV end-systolic volume index, Max LVWT, maximal LV wall thickness.

Figure 4 presented the correlations between LV FD parameters and functional parameters, as well as selected baseline parameters. All LV FDs showed a significant weak positive correlation with LV maximal wall thickness (maximal LVWT) (global FD *R* = 0.40, mean apical FD *R* = 0.37, maximal apical FD *R* = 0.40, mean basal FD *R* = 0.34, and maximal basal FD *R* = 0.38, all *P* < 0.001). Global FD, mean apical FD, and maximal apical FD were significantly correlated with LVEDV, LVESV, BMI, BSA, SBP, and DBP (all *P* < 0.05), while R values were small. LVEF was just negatively correlated with mean apical FD (*R* = −0.296, *P* < 0.05). Interestingly, mean basal FD and maximal basal FD were only correlated with age (mean basal FD, *R* = 0.200, *P* = 0.007; maximal basal FD, *R* = 0.206, *P* = 0.006), but not with global FD, mean, and maximal apical FD. Details are shown in Supplementary Table S1.

### Univariate and multivariable logistic regression analysis

In the univariate logistic regression analysis, traditional risk factors for HF (22), CMR parameters, and LV FD were included as exposure factors (Table 2). We found that LVEF, LVESV, LVEDVI, LVESVI, LVEDV, global FD [OR = 1.170, *P* < 0.001], maximal apical FD [OR = 1.070, *P* = 0.030], mean apical FD [OR = 1.121, *P* < 0.001], and mean basal FD [OR = 1.088, *P* = 0.036] were significantly associated with HF in univariate analysis. In contrast, maximal basal FD [OR = 1.034, *P* = 0.402] was not significantly associated.

**Table 2:** Univariate logistic regression analysis for hypertensive HF.

Characteristics	OR (95% CI)	*P*
Males	0.579 (0.253–1.324)	0.195
Age (years)	1.018 (0.997–1.040)	0.095
BMI (kg/m^2^)	0.992 (0.918–1.073)	0.848
BSA (m^2^)	0.355 (0.083–1.518)	0.162
SBP (mm Hg)	0.993 (0.981–1.006)	0.300
DBP (mm Hg)	0.994 (0.980–1.009)	0.464
Diabetes	0.944 (0.427–2.089)	0.887
Smoking history	0.935 (0.481–1.815)	0.842
Alcohol history	0.799 (0.398–1.601)	0.526
Dyslipidemia	1.205 (0.623–2.333)	0.580
Atrial fibrillation	1.528 (0.351–6.643)	0.572
LVEF, %	0.923 (0.891–0.956)	<0.001
LVEDV (mL)	1.012 (1.004–1.020)	0.002
LVESV (mL)	1.028 (1.014–1.041)	<0.001
SV (L/min)	0.991 (0.977–1.004)	0.175
Maximal LVWT (mm)	1.067 (0.955–1.193)	0.250
LVEDVI (mL/m^2^)	1.035 (1.017–1.053)	<0.001
LVESVI (mL/m^2^)	1.070 (1.039–1.101)	<0.001
Global FD	1.170 (1.068–1.280)	<0.001
Mean apical FD	1.121 (1.050–1.197)	<0.001
Maximal apical FD	1.070 (1.007–1.137)	0.030
Mean basal FD	1.088 (1.005–1.177)	0.036
Maximal basal FD	1.034 (0.956–1.119)	0.402

Fractal dimension is per 1% increase. Numbers in parentheses are 95% confidence intervals. The logistic regression analysis was conducted only for patients with hypertension (with and without HF).

OR, odds ratio; CI, confidence interval; BMI, body mass index; BSA, body surface area; SBP, systolic blood pressure; DBP, diastolic blood pressure; LVEF, LV ejection fraction; LVEDV, LV end-diastolic volume; LVESV, LV end-systolic volume; SV, stroke volume; FD, fractal dimension; LV, left ventricle; LVEDVI, LV end-diastolic volume index; LVESVI, LV end-systolic volume index; Maximal LVWT, maximal LV wall thickness.

Significant univariate parameters (*P* < 0.1) were included in the multivariable logistic regression analysis. After multivariable adjustment, mean basal FD [OR = 1.125 (95% CI: 1.010–1.253), *P* = 0.033] and mean apical FD [OR = 1.174 (95% CI: 1.012–1.362), *P* = 0.034] remained independently associated with HF (Table 3).

**Table 3:** Multivariable logistic regression analysis for hypertensive HF.

Characteristics	OR (95% CI)	*P*
LVEF (%)	0.915 (0.875–0.957)	<0.001
Age (years)	1.055 (1.023–1.087)	0.001
LVEDVI (mL/m^2^)	1.028 (1.004–1.052)	0.021
Mean apical FD	1.174 (1.012–1.362)	0.034
Maximal apical FD	0.921 (0.806–1.053)	0.227
Mean basal FD	1.125 (1.010–1.253)	0.033

LVEDV, LVESV, LVESVI, global FD were excluded from multivariate analysis because of VIF value >5. Fractal dimension is per 1% increase. Numbers in parentheses are 95% confidence intervals. The logistic regression analysis was conducted only for patients with hypertension (with and without HF).

OR, odds ratio; CI, confidence interval; LVEF, LV ejection fraction; LVEDV, LV end-diastolic volume; LVESV, LV end-systolic volume; LV, Left ventricular; FD, fractal dimension; LVEDVI, LV end-diastolic volume index; LVESVI, LV end-systolic volume index.

### Evaluation of the diagnostic value of fractal dimension for heart failure in hypertensive patients

The diagnostic value of FD for HF in hypertensive patients was assessed by incorporating FD parameters into diagnostic models for comparison (Table 4). In sequential nested logistic models, the traditional model based on conventional risk factors such as age, male, BMI, SBP, diabetes, and dyslipidemia was improved by the addition of traditional CMR predictors. Subsequently, the addition of LV FD to the model further enhanced discrimination, with an increase in the area under the curve (AUC) from 0.841 to 0.877 (DeLong's test, *P* < 0.05, Supplementary Table S2). Moreover, the predictive model that included FD demonstrated superior goodness-of-fit (−2 log-likelihood ratio test; *P* < 0.001, Supplementary Figure S1). Ultimately, these results indicate that LV FD contributes to the improvement of diagnostic models for patients in HF with hypertension compared with other conventional clinical and imaging risk factors.

**Table 4:** Predictive models of LV FDs for HF.

Models	AUC (95% CI)	*P*
Model 1	0.610 (0.518–0.702)	0.022
Model 2	0.818 (0.751–0.885)	<0.001
Model 3	0.841 (0.778–0.903)	<0.001
Model 4	0.877 (0.822–0.931)	<0.001

Model 1: Age + male + BMI + diabetes + dyslipidemia + SBP, Model 2: Model 1 + LVEF + LVEDV + LVESV + SV, Model 3: Model 2 + maximal LVWT, Model 4: Model 3 + global FD + mean apical FD + maximal apical FD + mean basal FD. Numbers in parentheses are 95% confidence intervals.

AUC, area under the curve; CI, confidence interval; BMI, body mass index; SBP, systolic blood pressure; LVEF, LV ejection fraction; LVEDV, LV end-diastolic volume; LVESV, LV end-systolic volume; LV, Left ventricular; FD, fractal dimension; LV, left ventricle; SV, stroke volume; FD, fractal dimension; Maximal LVWT, maximal LV wall thickness.

### Intra- and inter-observer reproducibility

Supplementary Table S3 displays intra- and inter-observer reproducibility. LV FD analysis demonstrated excellent intra- and inter-observer reproducibility (all ICC > 0.8).

## Discussion

This study performed fractal analysis to evaluate the diagnostic value of left ventricular endocardial trabecular complexity in patients with hypertensive heart failure. Our study identified that HTN patients without HF and HTN-HF patients both exhibited more complicated FD compared to the control group. Patients with HTN-HF showed significantly higher LV global FD, mean apical FD, maximal apical FD, and mean basal FD than HTN patients without HF. In addition, multivariable logistic regression analysis identified mean basal and apical FD as factors independently associated with HF. Furthermore, incorporating FD parameters into a diagnostic model significantly improved its discriminatory power and model fit compared to conventional clinical models.

Hypertension-related HF, a serious complication of HTN, poses a serious threat to patient survival due to its insidious nature, rendering early diagnosis challenging (1, 4, 23). Currently, echocardiography is a primary diagnostic tool for HF that enables the assessment of EF. However, in the early stages of HTN-HF, EF is often preserved, rendering the diagnosis of HF less reliable. Our study employed CMR-based fractal analysis to quantify the complexity of myocardial trabeculation, providing microstructural information that traditional imaging techniques fail to capture (7, 8). This approach offers a novel perspective for the early diagnosis of HF. The results demonstrated that left ventricular FD was significantly higher in hypertensive patients compared to healthy controls, and was even higher in those with heart failure. Notably, a previous multi-ethnic study of atherosclerosis revealed a positive correlation between HTN and FD (24). These results are consistent with our findings that FD is positively correlated with SBP and DBP in a dose-dependent manner, suggesting that FD shows promise as a dynamic marker for blood pressure-mediated myocardial remodeling. Furthermore, we found that sex was an independent factor associated with mean apical FD. Although the generalizability of this finding may be limited by the sex distribution in our cohort, it suggests potential sex-mediated differences in apical trabecular remodeling. Future prospective studies with sex-balanced designs are needed to validate this association and elucidate its underlying biological mechanisms.

Previous studies on myocardial trabeculation primarily focused on the volume and mass, as well as their relationships with LV volume, mass, and EF (25–29). However, in-depth research on the complexity of myocardial trabeculation remains relatively limited. With the advancement of technology, CMR has emerged as the gold standard for assessing the structure and function of both the atria and ventricles. Due to its high resolution and contrast, CMR enables the visualization of the complex endocardial trabecular structure. This capability offers a feasible and promising approach for investigating the complexity of endocardial trabeculation. Captur et al. (30) found that changes in LV trabeculation could be assessed using a semi-automated tool, with abnormal trabeculation often indicating disease. In this study, the complexity of LV trabeculation was higher in HTN patients with or without HF compared to the control group, and was even higher in HF patients, which is consistent with previous studies (14, 24, 26). As blood pressure increases, endocardial trabeculation is thought to undergo dilation and proliferation in response to pressure to maintain normal cardiac function (31). This process is inevitable and represents a variable phenotype of the disease state (24), supporting the view that the complexity of myocardial trabeculation may serve as a significant marker for the early diagnosis of heart failure. Our findings demonstrate the diagnostic utility of FD for discriminating heart failure within a hypertensive population, thereby establishing a foundation for investigating its prognostic potential. The translational value of this quantitative approach is further supported by research in other disease contexts. For instance, a semi-automated CMR tool developed by Tadros et al., which incorporated not only FD but also the trabecular mass ratio and composite surface ratio, successfully identified pediatric patients with left ventricular non-compaction who were at higher risk for adverse outcomes (32). Consequently, quantitative trabecular assessment in hypertensive heart disease holds promise to evolve from a diagnostic aid into a prognostic biomarker, a pivotal avenue for future prospective research.

The present study found that LV FDs progressively increased from the healthy control group to the HTN non-HF group and then to the HTN-HF group, with statistically significant differences observed between the HTN-HF and HTN non-HF groups. This gradient change suggests that the increased complexity of LV trabeculation may mirror the pathological progression from hypertension to HF. Mechanistic studies demonstrated that excessive trabecular proliferation is an adaptive remodeling in response to hemodynamic changes (31). On the one hand, myocardial trabeculation may serve to increase the surface area, which could assist in improving oxygen diffusion rate, particularly when coronary circulation is underdeveloped (33, 34). On the other hand, its fractal structure may help myocardial hypertrophy to optimize ventricular wall stress distribution, sustaining cardiac output when EF declines (34, 35). In this study, trabecular complexity increased as left ventricular ejection fraction decreased, further indicating the temporal and spatial relationship between increased trabecular complexity and ventricular function.

A recent study has also suggested that increased apical trabecular complexity is associated with slower LV systolic twist and diastolic untwist velocities (13), which may reflect changes in cardiac mechanics. Moreover, the structural complexity of myocardial trabeculae is closely linked to the arrangement and function of myocardial fibers (36), and changes in this complexity could impact overall cardiac contraction and relaxation, which may be linked to heart failure development. These findings highlight the unique value of CMR fractal analysis in detecting subclinical cardiac changes. Further investigations are needed to validate these findings. In the future, artificial intelligence algorithms could be combined with this method to establish a dynamic FD prediction model, offering a quantitative tool for risk stratification in hypertensive heart failure.

The study also demonstrated that global, mean/maximal apical FD were weakly positively correlated with LVEDV, LVESV, SV, and maximal LVWT, and also with BMI, BSA, SBP, and DBP. Additionally, only a weakly negative correlation was observed between LVEF and mean apical FD. Similar findings were reported in studies evaluating left ventricular non-compaction (37). These findings indicated that the complexity of myocardial trabeculae is correlated with cardiac function. Age showed a weak correlation with mean and maximal basal FD, implying that LV basal trabecular complexity may increase with age. This region-specific association was not observed in other ventricular segments. These results collectively suggest that structural changes in myocardial trabeculation are associated with a combination of various factors.

All LV FD parameters were included in univariate logistic regression analyses. The global FD, maximal and mean apical FD, mean basal FD, significantly differed between HTN-HF patients and HTN patients without HF. After multivariable regression analysis, mean basal and mean apical FD remained strong predictors of HF. Moreover, as demonstrated in several previous studies, trabecular complexity has shown good incremental prognostic value in a variety of cardiomyopathies (18, 38, 39). Therefore, further research is required to explore the potential prognostic value of LV FDs in HTN-HF patients.

In constructing the diagnostic model, combining the global FD, mean basal and apical FD, maximal basal and apical FD significantly enhanced its diagnostic performance. ROC curve analysis showed that the inclusion of these parameters improved discrimination, with an increase in the AUC from 0.671 in the baseline model to 0.877. These results suggest that LV FDs are associated with early cardiac structural and functional changes in HTN-HF patients and show promise as a valuable clinical diagnostic tool.

## Limitations

Nevertheless, the limitations of the present study should be acknowledged. First, its retrospective, single-center design limits causal inference and may introduce selection bias. Second, the modest sample size and absence of an external validation cohort affect the generalizability of our model. Finally, the cross-sectional nature of the analysis and lack of longitudinal follow-up prevent assessment of the prognostic value of trabecular complexity. Future prospective studies with larger, multi-center cohorts are needed to validate these findings and evaluate their clinical utility over time.

## Conclusion

In conclusion, CMR fractal analysis is a feasible technique for analyzing LV myocardial trabecular complexity in HTN-HF patients. The technique has demonstrated valuable incremental diagnostic utility for diagnosing hypertensive heart failure. Incorporating FD into the diagnostic models helps to improve diagnostic performance.

## Data Availability

The raw data supporting the conclusions of this article will be made available by the authors, without undue reservation.
