# Supplementary material for: Fractal analysis of left ventricular trabeculae in hypertensive patients with heart failure: a 3.0 T cardiac magnetic resonance study
Source: Front Cardiovasc Med. 2025 Nov 27;12:1697453. doi: 10.3389/fcvm.2025.1697453 (PMC12695737; doi:10.3389/fcvm.2025.1697453)
Supplement: Supplementary file 1 [file Datasheet1.pdf]

Supplementary Table 1 Correlations between LV FDs and LV functional parameters and clinical parameters.

Parameters	Global FD		Mean apical FD		Maximal apical FD		Mean basal FD		Maximal basal FD	
	r	p	r	p	r	p	r	p	r	p
**Age**	0.124	0.098	0.019	0.802	0.066	0.381	0.200	0.007	0.206	0.006
**LVEF**	-0.183	0.014	-0.296	< 0.001	-0.182	0.015	0.003	0.969	0.108	0.151
**BMI**	0.295	< 0.001	0.309	< 0.001	0.271	< 0.001	0.217	0.003	0.181	0.015
**SBP**	0.216	0.004	0.206	0.006	0.204	0.006	0.189	0.011	0.244	0.001
**DBP**	0.208	0.005	0.227	0.002	0.198	0.008	0.147	0.048	0.189	0.011
**BSA**	0.236	0.001	0.269	< 0.001	0.224	0.003	0.146	0.051	0.083	0.269
**Maximal LVWT**	0.403	< 0.001	0.366	< 0.001	0.403	< 0.001	0.340	< 0.001	0.380	< 0.001
**LVEDV**	0.317	< 0.001	0.388	< 0.001	0.294	< 0.001	0.161	0.031	0.061	0.413
**LVESV**	0.275	< 0.001	0.375	< 0.001	0.241	0.001	0.089	0.234	-0.025	0.742
**SV**	0.196	0.008	0.177	0.018	0.205	0.006	0.184	0.013	0.162	0.030
**LVEDVI**	0.259	< 0.001	0.327	< 0.001	0.244	0.001	0.118	0.113	0.040	0.592
**LVESVI**	0.241	0.001	0.345	< 0.001	0.216	0.004	0.055	0.459	-0.040	0.594

r correlation coefficient, FD Fractal dimension, BMI Body mass index, BSA Body surface area, SBP systolic blood pressure, DBP diastolic blood pressure, LVEF LV ejection fraction, LVEDV LV end-diastolic volume, LVESV LV end-systolic volume, SV Stroke volume, LV Left ventricular, LVEDVI LV end-diastolic volume index, LVESVI LV end-systolic volume index, Maximal LVWT maximal LV wall thickness.

Supplementary Table 2 DeLong’s test of predictive models for HF

Models	Difference of AUC	SE	95%CI	Z	P
**Model 1-Model 2**	-0.208	0.283	-0.306 – -0.111	-4.197	＜0.001
**Model 1-Model 3**	-0.231	0.280	-0.330 – -0.132	-4.570	＜0.001
**Model 1-Model 4**	-0.267	0.273	-0.366 – -0.168	-5.281	＜0.001
**Model 2-Model 3**	-0.022	0.253	-0.050 – -0.005	-1.595	0.111
**Model 2-Model 4**	-0.058	0.246	-0.101 – -0.016	-2.690	0.007
**Model 3-Model 4**	-0.036	0.241	-0.068 – -0.004	-2.173	0.030

Note:The AUC values between the groups were compared using the Delong test. AUC area under the curve, CI confidence interval, SE Standard error, βBeta coefficient, Z Z statistic. Model 1: Age+male+BMI+diabetes+dyslipidemia+SBP; Model 2: Model 1+LVEF+LVEDV+LVESV+SV; Model 3: Model 2+maximal LVWT; Model 4: Model 3+global FD+mean apical FD+maximal apical FD+mean basal FD. BMI Body mass index, SBP systolic blood pressure, LVEF LV ejection fraction, LVEDV LV end-diastolic volume, LVESV LV end-systolic volume, LV Left ventricular, FD fractal dimension, LV left ventricle, SV Stroke volume, FD fractal dimension, Maximal LVWT maximal LV wall thickness.

Supplementary Table 3 Intra- and inter-observer LV FD parameters

Parameters	Intraobserver		Interobserver	
	ICC	95%CI	ICC	95%CI
**Global FD**	0.965	0.928 - 0.982	0.977	0.948 – 0.989
**Mean apical FD**	0.972	0.950 - 0.985	0.983	0.965 – 0.991
**Maximal apical FD**	0.979	0.962 - 0.988	0.958	0.923 – 0.977
**Mean basal FD**	0.908	0.677 - 0.963	0.879	0.561 –0.952
**Maximal basal FD**	0.958	0.925 - 0.977	0.981	0.966 – 0.990

Note: Numbers in parentheses are 95% confidence intervals.CI confidence interval, FD fractal dimension, ICC intra-class correlation coefficient.

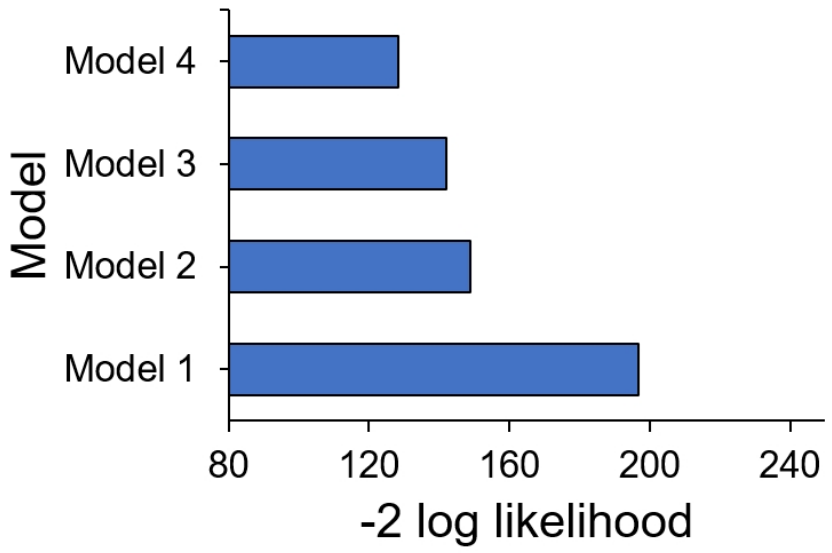

Supplementary Figure 1 Incremental prognostic value of LV FDs over conventional clinical and cardiac MRI risk factors. Model 1: Age+male+BMI+diabetes+dyslipidemia+SBP; Model 2: Model 1+LVEF+LVEDV+LVESV+SV; Model 3: Model 2+maximal LVWT; Model 4: Model 3+global FD+mean apical FD+maximal apical FD+mean basal FD. BMI Body mass index, SBP systolic blood pressure, LVEF LV ejection fraction, LVEDV LV end-diastolic volume, LVESV LV end-systolic volume, LV Left ventricular, FD fractal dimension, LV left ventricle, SV Stroke volume, FD fractal dimension, Maximal LVWT maximal LV wall thickness.
